# Heterotopic gastrointestinal cyst mimicking chronic cholecystitis: a case report

**DOI:** 10.1186/1752-1947-2-173

**Published:** 2008-05-22

**Authors:** Angel I Popkharitov, Maya V Gulubova, Angel D Dandov, Dimitar P Sivrev

**Affiliations:** 1 Department of Surgery, Medical Faculty, Thracian University, University Hospital, Stara Zagora, Bulgaria; 2 Department of General and Clinical Pathology, Medical Faculty, Thracian University, University Hospital, Stara Zagora, Bulgaria; 3 Department of Anatomy Medical Faculty, Thracian University, University Hospital, Stara Zagora, Bulgaria

## Abstract

**Introduction:**

Heterotopic gastric mucosa is described almost everywhere in the gastrointestinal tract, from the oral cavity to the rectum. The occurrence of heterotopic gastric tissue in the gallbladder is rare. A choristoma can be defined as a new growth developing from a displaced anlage not normally present in the anatomical site where it developed. We present an extremely uncommon case of a cyst (choristoma) attached to the gallbladder, which contained gastric and intestinal mucosa.

**Case presentation:**

A 33-year-old woman was hospitalized with clinical symptoms of chronic cholecystitis. The laboratory findings were within the normal range. Abdominal ultrasonography revealed a thickened gallbladder wall and a stone in the cystic duct was suspected. In the course of laparoscopic cholecystectomy, a cyst was visualized in the vicinity of the duct and the gallbladder neck. Microscopic examination of the removed cyst revealed evidence of gastric, duodenal and small-intestinal mucosa. The immunohistochemical study revealed many endocrine cells, which were positive for several endocrine cell markers such as chromogranin, serotonin, gastrin and so on. It can be inferred that the observed cyst had arisen from the foregut early in the development of the gastrointestinal tract.

**Conclusion:**

The presence of endocrine cells together with epithelial cells supports the hypothesis that these had developed simultaneously, and that the endocrine cells had probably supported the development of the epithelial cells by the release of hormones and growth factors. To the best of the authors' knowledge, this report is the first to report a gastrointestinal cyst choristoma with endocrine cells in the region of the cystic duct and gallbladder.

## Introduction

Heterotopic gastric mucosa is described almost everywhere in the gastrointestinal tract, from the oral cavity to the rectum [1]. The occurrence of heterotopic gastric tissue in the gallbladder is rare, although it has been reported by several authors [2-5].

A choristoma can be defined as a new growth developing from a displaced anlage (primordium or first gathering of embryonic cells) not normally present in the anatomical site where it has developed [3].

We present an extremely uncommon case of a cyst (choristoma) attached to the gallbladder, which contained gastric and intestinal mucosa.

## Case presentation

On 5 November 2001, a 33-year-old woman was admitted to hospital with a history of intermittent abdominal pain in the right upper abdominal quadrant, associated with nausea and vomiting. Past medical history revealed incidences of mild complaints of discomfort and pain in the same region radiating to the shoulder and the back. There was no previous history of jaundice.

The physical examination revealed a slight tenderness in the right upper abdomen. Murphy's sign was positive. The tumor markers were negative. The laboratory data were within the normal range.

Abdominal ultrasonography demonstrated a slightly thickened gallbladder wall and a stone in the cystic duct was suspected. We performed laparoscopic cholecystectomy based on the clinical diagnosis of chronic cholecystitis and cholelithiasis.

**Figure 1:**
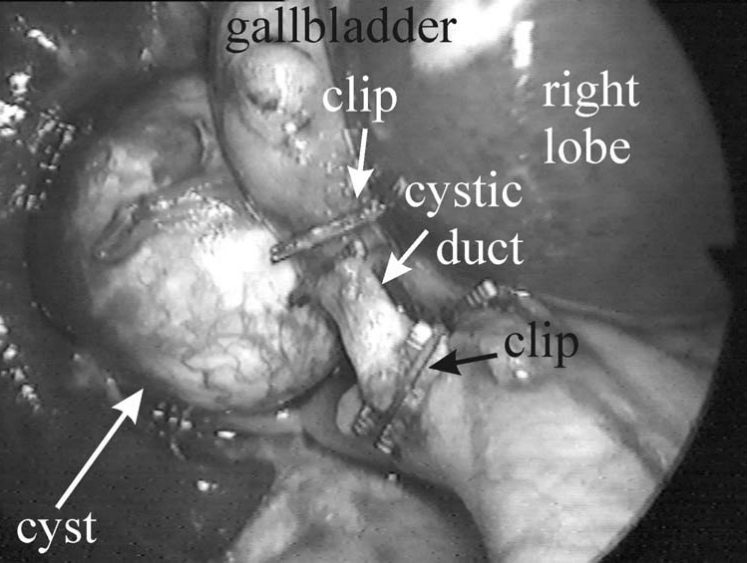
**Intra-operative image**. The cystic duct is dissected and doubly clipped. The gastrointestinal cyst is situated behind and attached to the gallbladder.

During laparoscopy, an oval cyst about 2 × 3 cm in size was visualized (Figure 1). It was situated in the vicinity of the cystic duct and the neck of the gallbladder and was intimately attached to its wall. The gallbladder itself and the cyst were wrapped in fine adhesions, attaching them to the duodenal wall. The gallbladder wall was slightly thickened and inflamed. Laparoscopic cholecystectomy and cystectomy were performed. No gallstones were found. When examined macroscopically, longitudinal resection of the cyst revealed no communication with the gallbladder. The cyst was tightly coalesced to the gallbladder wall. It was filled with a clear yellow fluid. The patient had an uneventful postoperative recovery and was discharged on the second postoperative day in good condition.

**Figure 2:**
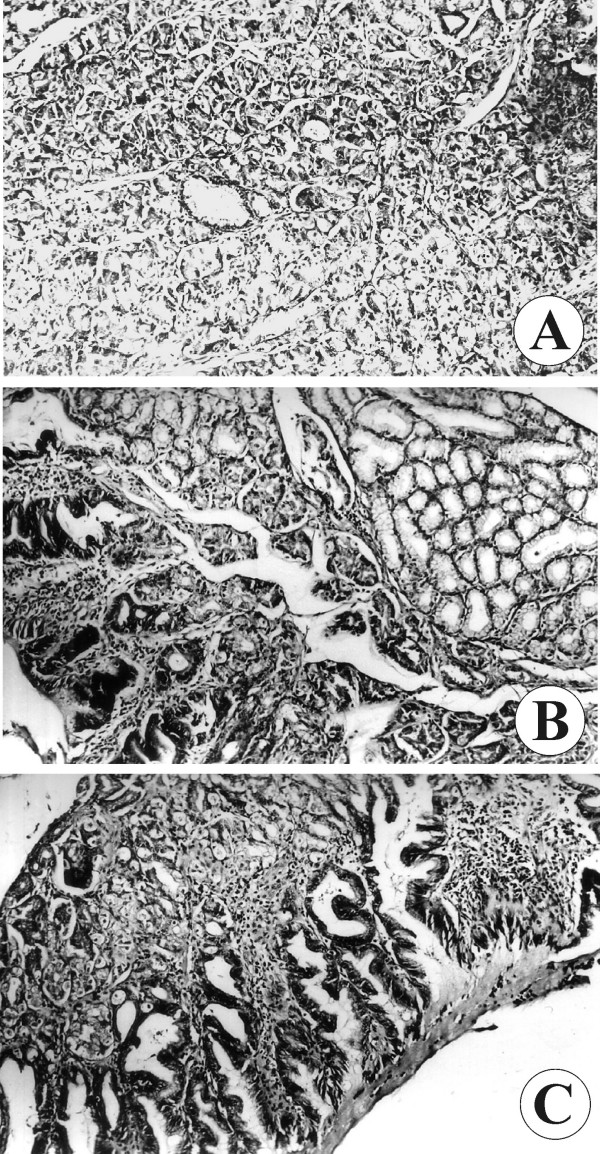
**The types of mucosa observed during microscopic examination**. (A) Fundic-type and (B) duodenal-type gastric mucosa; (C) intestinal-type villous mucosa.

**Figure 3:**
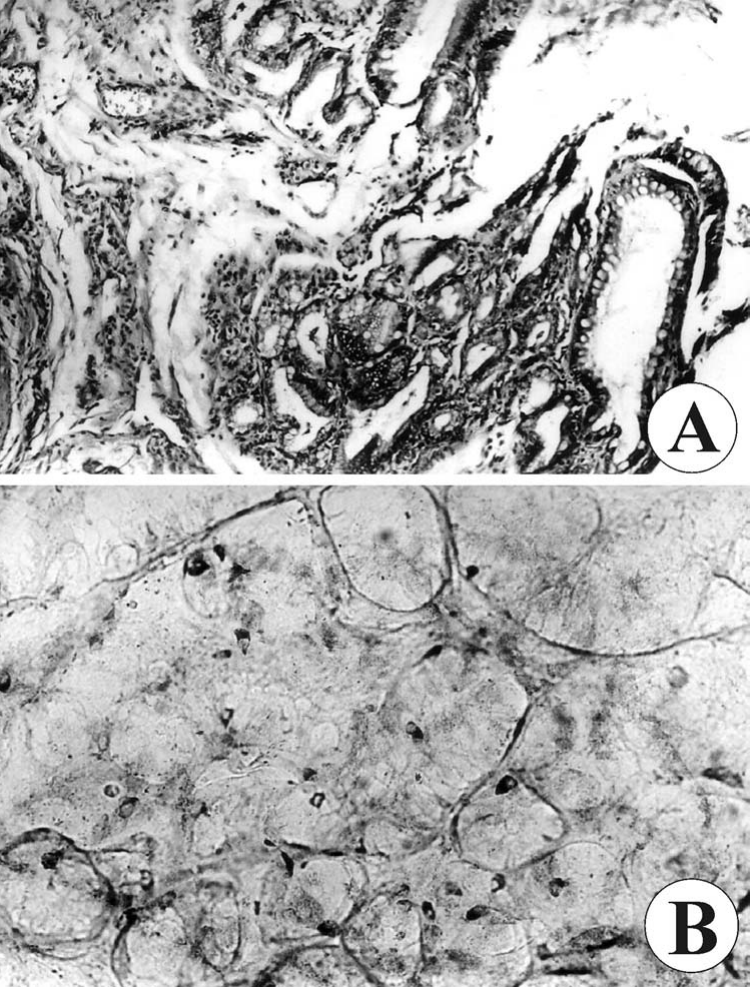
**The different types of cells observed during microscopic examination**. (A) Goblet cells and (B) serotonin-positive endocrine cells.

The microscopic examination showed that the cyst was flattened and contained mucosa from differing histological types. Gastric mucosa (fundic type; Figure 2A), duodenal mucosa with Brunner glands (Figure 2B) and intestinal mucosa with villi (Figure 2C) were observed. Parts of the mucosa containing cystic glands were chaotically intermingled with connective tissue stroma and small glands. Goblet cells were also seen (Figure 3A).

## Immunohistochemistry

The antibodies used were: rabbit antihuman chromogranin A (N1535), rabbit antihuman synaptophysin (N1566), mouse antihuman synaptophysin (U0037), rabbit antihuman somatostatin (N1551) and mouse antihuman serotonin (N1530), all obtained from DAKO A/S Denmark. The rabbit antihuman gastrin (PA019-5P), rabbit antihuman bombesin (PA062-5P), rabbit antihuman secretin (PA067-5P) and rabbit antihuman beta-endorphin (PA063-5P) were products of BioGenex Laboratories, San Ramon, CA, USA. The detection system used was DAKO LSAB^®^2 System, HRP (K0675), and DAKO^®^DAB Chromogen tablets (S3000) (DAKO A/S Denmark).

The cyst mucosa showed numerous endocrine cells located in the gastric glands (gastrin, somatostatin and serotonin), the duodenal glands (serotonin, somatostatin, secretin, gastrin and bombesin) and the small-intestinal glands (serotonin, somatostatin, secretin and bombesin). The endocrine cells were also positive for their typical markers chromogranin A, synaptophysin and beta-endorphin. They were dispersed chaotically in the mucosa, some were clustered and others distributed as single cells in the glands. Endocrine cells positive for serotonin are shown in Figure 3B.

## Discussion

A heterotopic gastrointestinal cyst is a rarely observed choristoma, composed of gastric and intestinal mucosa. It has been described mainly in the oral cavity [5], the mediastinum [6] and the retroperitoneum between the stomach and left adrenal gland [7], and is thought to be derived from misplaced embryonal residues. It is usually a cystic formation existing from birth, which is accidentally found in the thoracic or abdominal cavity. Histologically, it contains gastric and intestinal epithelium and sometimes also has bronchial mucosa [7].

In our case, a gastroenteric cyst was found in the vicinity of the cystic duct and did not come into contact with the biliary pathways or the duodenum. Histological examination showed that the cyst contained fundic-type gastric mucosa, duodenal mucosa and small intestinal mucosa with goblet cells.

The gastrointestinal tract and the accessory digestive organs develop from modifications of the primitive gut, which elongate and differentiate into the foregut, midgut and hindgut. The foregut includes the pharynx, mouth, esophagus, stomach, hepatic cells and bile ducts, pancreatic islands and the duodenum [8]. From our findings, it can be inferred that the observed cyst arose from the foregut very early in the development of the gastrointestinal tract. This is consistent with the gastric and intestinal mucosa found and the absence of hepatic and biliary tissue in the cyst lining.

Endocrine cells are widely distributed in the epithelial layer in organs that originate from the primitive gut [9]. The ontogeny of the gastrointestinal neuroendocrine system includes the simultaneous development of gut endocrine cells and neurons of the peripheral nervous system [10]. The first endocrine cells in the gut appear no later than the 10th week of gestation in both the stomach and the intestine [11]. Epithelial cells containing gastrin and somatostatin are found during the early period of gastric and small-intestinal development. The small-intestinal epithelium also contains bombesin-positive cells [12].

The clinical presentation of a heterotopic gastrointestinal cyst is non-specific and is associated with chronic cholecystitis. The clinical symptoms are generally biliary attacks, nausea and vomiting [13].

The most interesting finding of this report is that tissue from a common origin can be present in an uncommon anatomical position and mimic a completely different pathogenesis.

## Conclusion

From a surgical point of view, the presence of a gastroenteric cyst in the region of the gallbladder and biliary pathways should be recognized as a random formation that has to be differentiated from other tumor lesions.

In our case we observed the presence of a great variety of endocrine cells in the cyst mucosa, which can normally be detected in the stomach and intestine. To the best of the authors' knowledge, there are no other reports of the presence of endocrine cells in a gastric cyst. Their simultaneous occurrence in the cyst shows that the epithelial and endocrine cell components of the gastrointestinal mucosa develop at the same time, and the latter support the development of the former by the release of proper hormones and growth factors [10,11].

## Competing interests

The authors declare that they have no competing interests.

## Authors' contributions

AP conceived the idea for the study and made a substantial contribution to the sequence alignment and possible sources for the references, as well as drafting the definitive version of the manuscript. MG contributed to the interpretation of the histological section and immunochemistry study of the case. AD and DS made a substantial contribution to the embryologic section, as well as taking and interpreting the photographs. All authors edited and approved the final version of the manuscript.

## Consent

Written informed consent was obtained from the patient for publication of this case report and any accompanying images. A copy of the written consent is available for review by the Editor-in-Chief of this journal.
